# Stromal myofibroblasts in potentially malignant and malignant lesions of the oral cavity

**DOI:** 10.3892/ol.2014.2763

**Published:** 2014-12-03

**Authors:** PRISCILA CAMPIONI RODRIGUES, MÁRCIA CRISTINA DA COSTA MIGUEL, SIBELE NASCIMENTO DE AQUINO, FELIPE PAIVA FONSECA, ALAN ROGER DOS SANTOS SILVA, ADRIANA FRANCO PAES LEME, RICARDO D. COLETTA

**Affiliations:** 1 Department of Oral Diagnosis, School of Dentistry, State University of Campinas, Piracicaba, São Paulo 13414-018, Brazil; 2 Department of Dentistry, Federal University of Rio Grande do Norte, Natal, Rio Grande do Norte 59072-970, Brazil; 3 Brazilian Biosciences National Laboratory, National Center for Research in Energy and Materials, Campinas, São Paulo 13083-970, Brazil

**Keywords:** myofibroblast, potentially malignant oral lesions, oral squamous cell carcinoma, oral verrucous carcinoma

## Abstract

Previous studies have demonstrated that myofibroblasts in the adjacent stroma are involved in the development and progression of malignant tumors. The aim of this study was to investigate the involvement of myofibroblasts in the progression of oral squamous cell carcinomas (OSCCs) by determining myofibroblast density in potentially malignant and malignant oral lesions. A total of 69 potentially malignant oral lesions (leukoplakias with mild, moderate or severe dysplasia), 90 OSCCs (well-, moderately and poorly differentiated), eight oral verrucous carcinomas and 29 fibrous hyperplasias were examined for the presence of myofibroblasts using immunohistochemical detection of isoform α of smooth muscle actin. Myofibroblasts were not identified in the adjacent stroma of fibrous hyperplasias and potentially malignant oral lesions, whereas 59.8% of the oral carcinomas exhibited myofibroblasts in various densities. The density was significantly higher in moderately and poorly differentiated OSCCs when compared with well-differentiated tumors (P=0.04 and P=0.007, respectively). In verrucous carcinomas, the specific variant of well-differentiated OSCC, stromal myofibroblasts were not detected. The results of the present study demonstrated that immunodetection of myofibroblasts does not aid with the determination of the malignant transformation potential of oral dysplasias, although moderately and poorly differentiated tumors exhibited a significantly higher density of myofibroblasts. The results reinforce the hypothesis that myofibroblasts may contribute to oral tumorigenesis, indicating that verification and monitoring of such may serve as a putative marker of OSCC behavior.

## Introduction

Oral squamous cell carcinoma (OSCC) is the eighth most prevalent malignant neoplasm worldwide, and mortality rates are high in the majority of countries, with an overall five-year survival rate of <50% (1). OSCCs are usually diagnosed at an advanced stage; however, invasive diseases are often preceded by the presence of clinically identifiable potentially malignant lesions, including leukoplakias, erythroplakias and oral submucous fibrosis (2). Although the presence and severity of epithelial dysplasia has been associated with transformation into carcinoma, the mechanisms of progression are poorly understood, and the transformation rates are extremely variable (2,3).

Previous studies have indicated that specific alterations in the underlying connective tissue are associated with tumor development and progression (4,5). Tumor and stromal cells exchange cytokines, extracellular matrix proteins and enzymes that promote growth directly via stimulation of proliferation and survival, as well as invasion via local proteolysis of the extracellular matrix (6–8). Notably, previously, an increased density of myofibroblasts, also termed carcinoma-associated fibroblasts, in the stroma of OSCCs was found to correlate with lymph node metastasis and higher mortality (9–12). However, only a small number of studies have assessed the involvement of the subjadjacent connective tissue, particularly of myofibroblasts, on malignant transformation of potentially malignant oral lesions. In the present study, the density of myofibroblasts in potentially malignant and malignant lesions of the oral cavity was investigated to provide novel insights with regard to the involvement of myofibroblast cells in the development of OSCCs.

## Material and methods

### Tissue samples

This study included 29 cases of oral fibrous hyperplasia, 69 oral leukoplakias (of which 24 cases were histologically classified as mild epithelial dysplasia, 26 as moderate dysplasia and 19 as severe dysplasia), 90 OSCCs (35 well-differentiated, 39 moderately differentiated and 16 poorly differentiated) and eight oral verrucous carcinomas. All samples were obtained from patients aged >45 years. Samples were obtained from the Department of Oral Diagnosis, School of Dentistry, State University of Campinas (Piracicaba, Brazil), and new sections were cut from the paraffin blocks and stained with hematoxylin and eosin. Oral epithelial dysplasias and OSCCs were classified according to the World Health Organization grading system (13). This study was approved by the ethics committee of the School of Dentistry, State University of Campinas.

### Immunohistochemistry

Immunohistochemical staining was performed on 3-μm tissue sections using the avidin-biotin-peroxidase complex method. Briefly, sections were deparaffinized and dehydrated using a graded series of ethanol. Sections were then subjected to antigen retrieval with 0.01 M citrate buffer, pH 6.0 in an electric pressure cooker and incubated with 3% aqueous hydrogen peroxide for 15 min to quench endogenous peroxidase. Next, the sections were incubated with monoclonal mouse anti-human α-smooth muscle actin (α-SMA) antibody (1:400; Clone 1A4; Dako, Carpenteria, CA, USA), followed by the LSAB detection system (Dako). Reactions were developed by incubating the sections with 0.6 mg/ml 3,3′-diaminobenzidine tetrahydrochloride (Sigma-Aldrich, St. Louis, MO, USA) containing 0.01% H_2_O_2_ and counterstained with Mayer’s hematoxylin (Sigma-Aldrich). The exclusion of the primary antibody served as the negative control, whereas normal blood vessels were used as internal positive controls. The density of myofibroblasts was classified as negative, scanty or abundant as described by Kellermann *et al* (9), where tumors with 0% α-SMA-positive cells were classified as negative, if >1–<50% of the stromal myofibroblasts were α-SMA-positive tumors were classified as scanty, and abundant if >50% of the stromal cells were α-SMA-positive cells.

### Statistical analysis

Differences in the density of myofibroblasts between groups were analyzed using cross-tabulation and χ^2^ tests, and P<0.05 was considered to indicate a statistically significant difference (GraphPad Prism software, version 5.0; GraphPad Software, Inc., La Jolla, CA, USA.).

## Results

**Figure 1:**
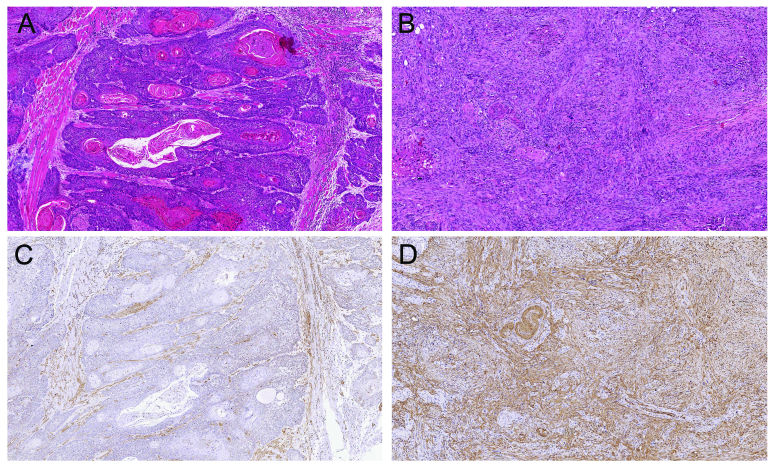
Histopathological features and detection of myofibroblasts in representative samples of OSCC. Histopathological features of (A) well-differentiated OSCC showing large nests of hyperchromatic cells with keratin pearls and (B) poorly differentiated OSCC, characterized by lack of cell keratinization, intense nuclear pleomorphism and features of atypical mitosis. (C) Immunohistochemical detection of myofibroblasts in the well-differentiated OSCC, in which the scanty presence of myofibroblasts surrounding the tumor nests was observed. (D) Abundant expression of myofibroblasts in poorly differentiated OSCC (stain, hematoxylin and eosin; magnification, ×100).

The density of myofibroblasts in the different oral lesions is shown in Table I. Myofibroblasts were not detected in oral fibrous hyperplasias or any of the 69 oral leukoplakias. Oral cancers exhibited positivity for myofibroblasts in 59.8% of the samples (Fig. 1). In well-differentiated OSCCs, the presence of myofibroblasts was classified as negative in 17 (48.6%) cases, scanty in 13 (37.1%) and abundant in five (14.3%). In moderately differentiated OSCCs nine (23.1%) cases were negative, 17 (43.6%) scanty and 13 (33.3%) abundant; whereas in poorly differentiated OSCCs, three (18.8%) cases were classified as negative, four (25.0%) as scanty and nine (56.2%) as abundant (Table I). In the samples of oral verrucous carcinomas, the presence of myofibroblasts was classified as negative in all cases (Table I). According to the histopathological differentiation, the density of myofibroblasts was significantly higher in moderately differentiated and poorly differentiated OSCCs, when compared with well-differentiated OSCCs (P=0.0402 and P=0.007, respectively). No significant differences were observed between moderately and poorly differentiated OSCCs (P=0.271).

## Discussion

Although a number of studies have indicated that the stroma is important in the development and progression of malignant tumors, the specific mechanisms associated with its activation and effects on regulation of the tumorigenesis remain unclear (4). One of the most evident processes in tumor stroma is the acquisition of myofibroblasts. The transdifferentiation and emergence of myofibroblasts has been considered a crucial event in tumorigenesis, and is mediated by growth factors and cytokines released by tumor cells (10,12,14,15). Myofibroblasts were originally identified in granulation tissues as modified fibroblasts with prominent rough endoplasmic reticulum and Golgi apparatus producing collagen, abundant myofilaments characterized by the presence of α-smooth muscle actin and fibronexus junctions (16). Later, it was shown that myofibroblasts may control a number of physiological and pathological events via the secretion of an extensive range of cytokines, growth factors, chemokines, hormones, neurotransmitters, inflammatory mediators, adhesion proteins and extracellular matrix proteins (7,17). Numerous studies have detected myofibroblasts in oral cancers (9–12,18–26); however, few studies have evaluated myofibroblasts in potentially malignant oral lesions (9,20,21,24–26) and in oral verrucous carcinomas (25).

In the present study, myofibroblasts were not found in oral fibrous hyperplasias or any of the 69 potentially malignant oral lesions. Previous studies did not identify myofibroblasts in oral normal mucosa or oral leukoplakias with dysplasia, which highlights the importance of the molecular crosstalk between stromal elements and tumor cells during invasion of the connective tissue for the emergence of myofibroblasts (9,21,26). However, a small number of studies identified myofibroblasts in oral dysplasias (20,24,25). Vered *et al* (20) analyzed 11 samples classified as mild dysplasia and 12 as moderate/severe dysplasia by directly counting α-SMA-stained cells, and identified sparse stromal myofibroblasts in the premalignant lesions with a mean percentage of ~1%, although the number of positive samples was not reported. Seifi *et al* (24) used the same scoring system that was applied in the present study and identified myofibroblasts in 4/18 (22.2%) epithelial dysplasia samples; however, this study did not grade the epithelial dysplasias. Chaudhary *et al* (25) reported that 7/15 (46.6%) cases of high-risk epithelial dysplasia (moderate and severe) exhibited myofibroblasts in various intensities, whereas all low risk dysplasias (hyperplasia and mild) were negative for myofibroblasts. However, in all three studies (20,24,25) the immunohistochemical images provided by the authors were not sufficient to distinguish smooth muscle cells from blood vessels of myofibroblasts or stromal reaction due to superficially invasive OSCC. The present study, which had the largest sample size when compared with previous studies, indicated that myofibroblasts are not associated with the transformation process of potentially malignant lesions of the oral cavity.

The present study also demonstrated that myofibroblasts were frequently identified in the stroma of invasive OSCCs. However, myofibroblasts were not detected in oral verrucous carcinoma, which is consistent with the hypothesis that oral verrucous carcinoma is a form of well-differentiated squamous cell carcinoma with specific clinical and histological features, including slow growth and no invasive potential, which is unlikely to metastasize (31). This reinforces the hypothesis that interactions between tumor cells and tumor microenvironment are important for oral carcinogenesis. Myofibroblasts in OSCCs may exert an active role in disease progression via autocrine effects on tumor stroma and paracrine effects on malignant epithelial cells through tumor-stromal interactions (10,27–29). The neoplastic changes that occur in the epithelium are followed by changes in the stroma surrounding tumor cells, which promote the differentiation of fibroblasts into myofibroblasts (30). Furthermore, a previous study demonstrated that transforming growth factor-β1, which is released by OSCC cells, was responsible for oral fibroblast to myofibroblast transdifferentiation in an experimental model (10). The presence of myofibroblasts was significantly higher in moderately and poorly differentiated OSCCs when compared with that of well-differentiated OSCCs. These results may indicate that the loss of cellular differentiation affects the number of myofibroblasts in the tumor stroma (22,25).

In conclusion, the results of this study indicated that myofibroblasts are not associated with potentially malignant oral lesions; however, moderately and poorly differentiated OSCCs exhibited a higher density of myofibroblasts when compared with well-differentiated tumors. The prognostic value of tumor differentiation in OSCC is unclear and, thus, further studies with larger patient cohorts are required to confirm the association between increased myofibroblast density, loss of differentiation and the biological behavior of tumors.

## Figures and Tables

**Table I:** Density of myofibroblasts in fibrous hyperplasias, potentially malignant oral lesions (leukoplakias with histologically confirmed dysplasia), OSCCs and oral verrucous carcinomas.

	Density of myofibroblast		
	
	Negative, n (%)	Scanty, n (%)	Abundant, n (%)
Fibrous hyperplasia	29 (100)	0 (0)	0 (0)
Leukoplakia with mild dysplasia	24 (100)	0 (0)	0 (0)
Leukoplakia with moderate dysplasia	26 (100)	0 (0)	0 (0)
Leukoplakia with severe dysplasia	19 (100)	0 (0)	0 (0)
Well-differentiated OSCC^a^,^b^	17 (48.6)	13 (37.1)	5 (14.3)
Moderately differentiated OSCC^c^	9 (23.1)	17 (43.6)	13 (33.3)
Poorly differentiated OSCC	3 (18.8)	4 (25.0)	9 (56.2)
Oral verrucous carcinoma	8 (100)	0 (0)	0 (0)

a P=0.042, negative, scanty and abundant percentage of myofibroblasts in well-differentiated OSCC compared with moderately differentiated OSCC;

b P=0.007, negative, scanty and abundant percentage of myofibroblasts in well-differentiated OSCC compared with poorly differentiated OSCC;

c P=0.271, negative, scanty and abundant in moderately differentiated OSCC compared with poorly differentiated OSCC.

OSCC, oral squamous cell carcinoma.

## References

[1] Warnakulasuriya S (2010). Living with oral cancer: Epidemiology with particular reference to prevalence and life-style changes that influence survival. Oral Oncol.

[2] Napier SS, Speight PM (2008). Natural history of potentially malignant oral lesions and conditions: an overview of the literature. J Oral Pathol Med.

[3] Hsue SS, Wang WC, Chen CH (2007). Malignant transformation in 1458 patients with potentially malignant oral mucosal disorders: a follow-up study based in a Taiwanese hospital. J Oral Pathol Med.

[4] Hanahan D, Coussens LM (2012). Accessories to the crime: functions of cells recruited to the tumor microenvironment. Cancer Cell.

[5] Alitalo A, Detmar M (2012). Interaction of tumor cells and lymphatic vessels in cancer progression. Oncogene.

[6] De Wever O, Mareel M (2003). Role of tissue stroma in cancer cell invasion. J Pathol.

[7] Desmouliere A, Guyot C, Gabbiani G (2004). The stroma reaction myofibroblasts: a key player in the control of tumor cell behavior. Int J Dev Biol.

[8] De Wever O, Demetter P, Mareel M, Bracke M (2008). Stromal myofibroblasts are drivers of invasive cancer growth. Int J Cancer.

[9] Kellermann MG, Sobral LM, da Silva SD (2007). Myofibroblasts in the stroma of oral squamous cell carcinoma are associated with poor prognosis. Histopathology.

[10] Kellermann MG, Sobral LM, da Silva SD (2008). Mutual paracrine effects of oral squamous cell carcinoma cells and normal oral fibroblasts: Induction of fibroblast to myofibroblast transdifferentiation and modulation of tumor cell proliferation. Oral Oncol.

[11] Bello IO, Vered M, Dayan D (2011). Cancer-associated fibroblasts, a parameter of the tumor microenvironment, overcomes carcinoma-associated parameters in the prognosis of patients with mobile tongue cancer. Oral Oncol.

[12] Marsh D, Suchak K, Moutasim KA (2011). Stromal features are predictive of disease mortality in oral cancer patients. J Pathol.

[13] Gale N, Pilch BZ, Sidransky D, El-Naggar AK, Westra W, Califano J, Johnson N, MacDonald DG, Barnes L, Eveson JW, Reichart P, Sidransky D (2005). Epithelial precursor lesions (Oral cavity and oropharynx). World Health Organization Classification of Tumours. Pathology and Genetics of Head and Neck Tumours.

[14] Baglole CJ, Ray DM, Bernstein SH (2006). More than structural cells, fibroblasts create and orchestrate the tumor microenvironment. Immunol Invest.

[15] Thode C, Jørgensen TG, Dabelsteen E, Mackenzie I, Dabelsteen S (2011). Significance of myofibroblasts in oral squamous cell carcinoma. J Oral Pathol Med.

[16] Gabbiani G, Ryan GB, Majno G (1971). Presence of modified fibroblasts in granulation tissue and their possible role in wound contraction. Experientia.

[17] Powell DW, Adegboyega PA, Di Mari JF, Mifflin RC (2005). Epithelial cells and their neighbors I. Role of intestinal myofibroblasts in development, repair, and cancer. Am J Physiol Gastrointest Liver Physiol.

[18] Barth PJ, Schenck ZU, Schweinsberg T, Ramaswamy A, Moll R (2004). CD34+ fibrocytes, alpha-smooth muscle antigen positive myofibroblasts and CD117 expression in the stroma of invasive squamous cell carcinoma of the oral cavity, pharynx, and larynx. Virchows Arch.

[19] Kojc N, Zidar N, Vodopivec B, Gale N (2005). Expression of CD34, alpha-smooth muscle actin, and transforming growth factor beta1 in squamous intraepithelial lesions and squamous cell carcinoma of the larynx and hypopharynx. Hum Pathol.

[20] Vered M, Allon I, Buchner A, Dayan D (2009). Stromal myofibroblasts accompany modifications in the epithelial phenotype of tongue dysplastic and malignant lesions. Cancer Microenviron.

[21] Etemad-Moghadam S, Khalili M, Tirgary F, Alaeddini M (2009). Evaluation of myofibroblasts in oral epithelial dysplasia and squamous cell carcinoma. J Oral Pathol Med.

[22] Kawashiri S, Tanaka A, Noguchi N (2009). Significance of stromal desmoplasia and myofibroblast appearance at the invasive front in squamous cell carcinoma of the oral cavity. Head Neck.

[23] Vered M, Dobriyan A, Dayan D (2010). Tumor-host histopathologic variables, stromal myofibroblasts and risk score, are significantly associated with recurrent disease in tongue cancer. Cancer Sci.

[24] Seifi S, Shafaei S, Shafigh E, Sahabi SM, Ghasemi H (2010). Myofibroblast stromal presence and distribution in squamous epithelial carcinomas, oral dysplasia and hyperkeratosis. Asian Pac J Cancer Prev.

[25] Chaudhary M, Gadbail AR, Vidhale G (2012). Comparison of myofibroblasts expression in oral squamous cell carcinoma, verrucous carcinoma, high risk epithelial dysplasia, low risk epithelial dysplasia and normal oral mucosa. Head Neck Pathol.

[26] De-Assis EM, Pimenta LGGS, Costa-e-Silva E, Souza PEA, Horta MCR (2012). Stromal myofibroblasts in oral leukoplakia and oral squamous cell carcinoma. Med Oral Patol Oral Cir Bucal.

[27] Lewis MP, Lygoe KA, Nystrom ML (2004). Tumour-derived TGF-beta1 modulates myofibroblast differentiation and promotes HGF/SF-dependent invasion of squamous carcinoma cells. Br J Cancer.

[28] Sobral LM, Bufalino A, Lopes MA (2011). Myofibroblasts in the stroma of oral cancer promote tumorigenesis via secretion of activin A. Oral Oncol.

[29] Hinsley EE, Kumar S, Hunter KD, Whawell SA, Lambert DW (2012). Endothelin-1 stimulates oral fibroblasts to promote oral cancer invasion. Life Sci.

[30] Bremnes RM, Dønnem T, Al-Saad S (2011). The role of tumor stroma in cancer progression and prognosis: emphasis on carcinoma-associated fibroblasts and non-small cell lung cancer. J Thorac Oncol.

[31] Alkan A, Bulut E, Gunhan O, Ozden B (2010). Oral verrucous carcinoma: a study of 12 cases. Eur J Dent.

